# The Value of Removing Daily Obstacles via Everyday Problem-Solving Theory: Developing an Applied Novel Procedure to Increase Self-Efficacy for Exercise

**DOI:** 10.3389/fpsyg.2013.00020

**Published:** 2013-01-29

**Authors:** Daniele Artistico, Angela Marinilli Pinto, Jill Douek, Justin Black, Lina Pezzuti

**Affiliations:** ^1^Department of Psychology, Baruch College, City University of New YorkNew York, NY, USA; ^2^University of Rome “La Sapienza,”Rome, Italy

**Keywords:** self-efficacy, physical activity, everyday problem-solving theory, everyday problem-solving training, idiosyncratic methods

## Abstract

The objective of the study was to develop a novel procedure to increase self-efficacy for exercise. Gains in one’s ability to resolve day-to-day obstacles for entering an exercise routine were expected to cause an increase in self-efficacy for exercise. Fifty-five sedentary participants (did not exercise regularly for at least 4 months prior to the study) who expressed an intention to exercise in the near future were selected for the study. Participants were randomly assigned to one of three conditions: (1) an Experimental Group in which they received a problem-solving training session to learn new strategies for solving day-to-day obstacles that interfere with exercise, (2) a Control Group with Problem-Solving Training which received a problem-solving training session focused on a typical day-to-day problem unrelated to exercise, or (3) a Control Group which did not receive any problem-solving training. Assessment of obstacles to exercise and perceived self-efficacy for exercise were conducted at baseline; perceived self-efficacy for exercise was reassessed post-intervention (1 week later). No differences in perceived challenges posed by obstacles to exercise or self-efficacy for exercise were observed across groups at baseline. The Experimental Group reported greater improvement in self-efficacy for exercise compared to the Control Group with Training and the Control Group. Results of this study suggest that a novel procedure that focuses on removing obstacles to intended planned fitness activities is effective in increasing self-efficacy to engage in exercise among sedentary adults. Implications of these findings for use in applied settings and treatment studies are discussed.

## Introduction

Regular physical activity is associated with numerous positive health outcomes such as reduced risk of cardiovascular disease, diabetes, and increased quality of life (Colcombe et al., 2004; Hamman et al., 2006; Warburton et al., 2006; Flöel et al., 2009; Reddigan et al., 2011). Despite the known benefits of exercise, many people struggle to engage in and maintain a regular exercise routine. Less than 25% of Americans aged 15 and older exercise regularly (Bureau of Labor Statistics, 2009), while many other individuals perceive physical activity to be a psychological challenge (Walcott-McQuigg and Prohaska, 2001). When younger adults are interviewed about the obstacles that stop them from exercising, some refer to physical barriers such as lack of transportation to the gym, while the large majority report psychological barriers, such as lack of motivation (Dishman et al., 2009).

Research has shown that success with initiating and maintaining a physical activity program is related to one’s self-efficacy perceptions, or one’s perceived ability to overcome self-reported obstacles to exercise (Bandura, 1997; Dishman et al., 2009; Doerksen et al., 2009; Prohaska et al., 2000). It would seem then, that increasing self-efficacy for exercise may be an effective vehicle for facilitating initiation and maintenance of physical activity.

This paper aims to demonstrate that the psychological underpinnings of the perceived self-efficacy for physical activity are related to people’s ability to overcome their self-reported obstacles to exercise. In so doing, we capitalized on recent problem-solving theoretical advances and applications (Allaire and Marsiske, 2002; Artistico et al., 2003, 2010, 2011; Pezzuti et al., 2009) and developed a novel experimental procedure to use in the context of physical activity. Specifically we addressed the challenges faced by individuals entering into an exercise routine by providing a structured problem-solving analysis for each obstacle that could interfere with exercise adherence.

### Increasing self-efficacy for exercise: An everyday problem-solving novel procedure

Self-efficacy is a cognitive mediator in the individual’s ability to overcome setbacks, challenges, and obstacles (Bandura, 1986; Caprara and Cervone, 2000). Perceptions of self-efficacy contribute to human adjustment and achievement in several cognitive and emotional domains (Bandura and Cervone, 1983; Bandura, 1986). To foster self-efficacy, Bandura (1997) recommended that investigators carefully design research that examines the determinants of the task that needs to be accomplished (in our case to remove obstacles that interfere with adherence to physical activities). Indeed, it has been convincingly shown that the most powerful source of performance is mastery of experiences (Bandura, 1977, 1997). In the domain of exercise, this could mean that individuals should first be able to “master” what stops them from exercising. The uniqueness of perceived obstacles is also relevant because one’s self-efficacy can be explained by idiosyncratic patterns of activation between self-knowledge and appraisals of the obstacles to be solved (Cervone, 2004).

Specifically, one needs to analyze the factors that undermine self-efficacy for exercise from the individual perspective. Research has demonstrated that a perceived lack of time, energy, sense of inability to exercise, and lack of social support from family or friends may differentially impact individuals (Dishman et al., 2004, 2009). A lack of problem-solving skills will lead to self-doubt and impede the formation of a robust sense of self-efficacy.

When people are committed to partaking in health activities, they may fail to act on their intentions because of situational factors. Among those situational factors are social, interpersonal, and intra-personal everyday problems. By training individuals to generate strategies for solving such everyday problems, the intention is to facilitate their daily adherence to exercise and reduce attrition from exercise programs. This is consistent with systematic research that demonstrates the way in which people who wish to exercise commonly confront everyday problems that impede their intended pursuits (Lee et al., 2008; Roessler and Ibsen, 2009).

Our goal is to show that young adults’ participation in exercise activity may hinge on their ability to solve everyday problems. Here we are mainly concerned with issues of perceived self-efficacy for exercise. We studied the interplay between everyday problem-solving and self-efficacy in the context of physical exercise. This is novel in the literature as no prior research has established a causal relationship between self-efficacy, exercise, and everyday problem-solving. We sought to improve everyday problem-solving skills first by having participants identify obstacles and solutions to their individual exercise-related problems, and second by encouraging them to consider additional solutions via idiographic experimental procedures. Gains in people’s ability to solve everyday problems are expected to increase self-efficacy for exercise. This notion was driven by previous research on everyday problem-solving which is extensively reviewed below (e.g., Reitman, 1964; Simon, 1973; Allaire and Marsiske, 2002; Artistico et al., 2003, 2010; Blanchard-Fields, 2007; Pezzuti et al., 2009).

### Everyday problem-solving theory

Everyday problem-solving ability is one’s capacity to overcome day-to-day obstacles. Researchers have focused on three intertwined elements in framing the study of everyday problem-solving: solution generation, problem-solving space, and the problem’s root cause. Solution generation refers to the cognitive process of conceptualizing and choosing obstacle-relevant strategies. The problem-solving space (Reitman, 1964; Simon, 1973) contains an everyday problem’s fundamental elements, the comprehension of which is required for effective solution generation. The root cause or causes of an everyday problem refer to the events or situations that acted as primary determinants of the problem-space. Below we briefly discuss these three elements in greater detail.

#### Solution generation

The ability to aptly identify an increasing number of strategies or solutions to a specific problem is central to everyday problem-solving (Allaire and Marsiske, 2002; Artistico et al., 2003, 2010). In complex and dynamic environments the exact level of success that will be achieved by a given strategy cannot be known. Inherent uncertainties in the environment in which a problem might take place (e.g., work, home, recreational settings), make it impossible to know which strategy will prove to be the most effective for a given decision maker. To the extent that one is able to generate multiple solutions to everyday problems, there will be viable alternatives available in case a strategy fails. One’s ability to solve everyday problems is a rather malleable cognitive ability. Indeed, researchers have previously demonstrated that it is possible to increase individuals’ solution generation ability (Pezzuti et al., 2009) by helping the problem-solver to address gaps in the problem-solving space.

#### The problem-solving space

The “problem-solving space” is a concept that was originally introduced by Reitman (1964) and Simon (1973) in order to guide our understanding of the underlying elements of an everyday problem. The problem-solving space is defined by three elements: the initial state, the means, and the final state. Everyday problems are considered ill-defined when one of the three elements is missing or not stipulated in the formulation of the problem (Allaire and Marsiske, 2002). Problem-space theory (Simon, 1973) proposes that in order to solve ill-defined problems, people must first fill in the gaps in their mental representation of the problem-space. In many cases the initial state is ambiguous and the means by which the final state is reached can be multiple. For example, if someone wishes to overcome feelings of loneliness, social contact may be increased in a variety of ways. However, if the lonely individual were to reflect on and identify the antecedents of those feelings of loneliness (e.g., he just moved to a different city), then he or she would be able to more aptly direct solution generation (e.g., increasing time spent in public or private events in the new city).

#### The root cause of the problem

The example above demonstrates the importance of root cause identification in the problem-solving process. The problem-solver can better define the problem-space when he or she understands the problem’s root causes. Such comprehension allows descriptions of the problem’s nature beyond those available from a consideration of its surface-level features. Those root-level descriptions fill gaps in mental representations of the initial and final states of a problem. Solutions (i.e., the *means*) flourish at each deeper level of definition. The optimal problem-solver will be able to identify the sub-goals or elements for everyday problems, allowing him or her to generate and propose alternative types of solutions to the problem according to its underlying elements (Allaire and Marsiske, 2002).

When using everyday problem-solving theory, investigators should provide individuals with a large number of problem-solving strategies alongside plausible root causes of the problem, thereby fostering connections between root causes and solutions. Further, because the intention to exercise is often correlated with perception of social support, and inversely related to the amount of perceived stress (Doerksen et al., 2009), a careful study design should include individual difference measures of both perceived stress and social support (Prohaska et al., 2000). Aspects of perceived self-knowledge not directly relevant to the challenges at hand can impact everyday problem-solving ability (Cervone, 2004).

### Aims

Generating dynamic solutions to everyday problems and identifying root causes of problems should help participants reduce the impact of obstacles that interfere with regular exercise activities by increasing their sense of self-efficacy. We expect that newly acquired problem-solving strategies attained via our brief experimental procedure will significantly enhance self-efficacy perceptions. Specifically, we hypothesized an interaction between the type of condition (experimental) and the time of the assessment (after the treatment) on self-efficacy for exercise. We tested this main hypothesis experimentally, after controlling for the effects of perceived stress and social support.

## Materials and Methods

### Participants

Participants were undergraduate students from a large university in the Mid-Atlantic U.S. who volunteered for the study in exchange for partial research credit toward their course requirements. The study was advertised online through a college subject pool website and specifically targeted sedentary college students. Inclusion criteria for the study were: not currently engaged in a regular exercise program, tried but failed to do so in the past 4 months, and intention to begin an exercise program in the near future.

Sixty-five subjects provided informed consent to participate in the study, but only 55 completed the study procedures (one participant misunderstood the selection criteria, whereas the other nine participants did not return for the second session of the study). The sample size was compared with the power analysis on research linking everyday problem-solving and self-efficacy (Artistico et al., 2003). The power analysis (0.80) suggested by Keppel (1991) indicated that a sample of 50 subjects would produce a statistically significant effect size.

The sample was ethnically (30.9% Hispanic or Latino) and racially (33.3% Asian, 31.5% Caucasian, 9.3% Black or African American, 25.9% other or did not report) diverse. The sample was composed of male (40%) and female (60%) younger adults (*M* = 21.38, SD = 4.48). The study groups (see Procedures immediately below) did not differ significantly by sex (χ^2^ = 2.71, *p* = 0.26), ethnicity (χ^2^ = 0.98, *p* = 0.61), or race (χ^2^ = 6.17, *p* = 0.41).

### Procedures

Participants who met study inclusion criteria attended the first laboratory session lasting approximately 30 min in which they signed informed consent documents, underwent a screening procedure to confirm that (1) they were not engaged in an exercise program and (2) had intention to exercise, and completed baseline questionnaires. Participants returned to complete a second laboratory session 1 week later. During the second laboratory session, participants were randomly assigned to one of three conditions: Experimental Group (*n* = 18), Control Group with Problem-solving Training (*n* = 22), or Control Group (without problem-solving training; *n* = 15). The training procedure for the Experimental Group and Control Group with Problem-solving Training lasted approximately 45 min (see Intervention Conditions section); following the training procedure participants completed a second set of questionnaires, which took approximately 15 min. Participants in the Control Group returned to the laboratory for Session 2 only to complete study questionnaires.

To ensure privacy and to create a tranquil environment for the study, participants worked individually in a lab facility. The experimenter was in the background to prompt participants about the next task. Although the study instructions stressed to the participants to take a break if needed, the experimenter did not notice that any breaks were taken. Assessments were presented via computer (measures) or on paper (the training sessions). Once participants completed the second session, they were debriefed about the purpose of the study. The Institutional Review Board of the City University of New York approved the study procedures.

#### Intervention conditions

##### Experimental group

Participants in this group received tailored materials which were designed to specifically address challenges with exercise (Part A). All participants were asked to list their three primary reasons for not exercising. Participants then engaged in the following assignments: (a) generate alternative solutions to ideographically identified obstacles, (b) identify potential root causes of the obstacles, (c) connect solutions with the root causes of the obstacles, and (d) recall the solutions generated. These are discussed in more detail below.

The goal of the solution generation phase (Part A) was to compose a tailored list of potentially viable solutions for each participant, as has been done in prior work on everyday problem-solving (cf. Pezzuti et al., 2009; Artistico et al., 2010). The first strategy was to look at failed prior attempts to solve the problem, as reported by the participant. This was important because we did not want to activate heuristics that would prime failure. The second strategy was to use a “thinking-out-loud” procedure in which we asked a small group of subject matter experts to generate as many solutions as possible. The third strategy was to integrate information obtained from specialized literature. Specifically, we consulted Medline and PsycInfo databases along with some self-help websites in order to offer participants as many of the most viable solutions as possible.

*Type of solution validation testing*. In the current study, we offered solutions to participants that represented a mix between interpersonal or instrumental ways to solve everyday problems. Because in the everyday problem-solving literature (Blanchard-Fields, 2007), the way one approaches a problem or problem-solving style (interpersonal or instrumental) has an impact on solution generations, we tested such impact by using one independent sample of 93 undergraduate students coming from a similar population. The specific goal was to see if participants preferred interpersonal or instrumental solutions (independent variable) to problems related to physical exercise. Each participant rated 12 solutions. Neither the type of solution (interpersonal or instrumental) nor the participants’ preference between an interpersonal versus an instrumental way to solve problems produced any significant effects (*F*_5;86_ = 0.34) on perceived self-efficacy, as assessed by a standard scale on self-efficacy and exercise (cf. Plotnikoff and Higginbotham, 2002).

The final product was a bank of solutions, which was mapped ideographically to each participant’s problem according to two principles that we specifically developed for this procedure. The first principle is the exclusion of failed strategies (this could vary between individuals), and the second is independence of strategies (this could vary within individuals). According to the first principle, we would exclude strategies that participants have already tried in the past. Hence, two participants with the same problem would not necessarily be provided the same solutions. However, for experimental consistency, each participant received the same number of total solutions (33 solutions for each of the three major problems) yielding a total of 99 solutions. We also allowed participants to generate solutions via a write-in response in addition to the ones we provided. No participant used the write-in space to offer additional solutions. To apply the second principle (independence of strategies), we made sure that no two sets of solutions were identical.

The goal of the second phase (Part B) was to identify the potential root causes of the three obstacles to exercise. We employed the same three strategies and two guiding principles discussed above to identify four major root causes for each problem, yielding a total of 12 root causes.

The goal of the third phase (Part C) was for participants to complete a written exercise designed to integrate Parts A and B. We asked our participants to assign one or more solutions from Part A to each of the root causes identified in Part B, which were the causes that were relevant to them.

The goal of the fourth phase (Part D), was for participants to “think back” to the problem they had previously worked on, and to remember as many solutions as possible for that specific problem, without looking back at their previous pages. The task was to say out loud as many solutions as possible that were recalled (or to write them down on a blank paper).

In doing so, the novel experimental procedure process included two components that have proven important in cognitive-behavioral problem-solving interventions: (1) The process of solution generation, that is, the ability to find many effective ways to solve the same problem and (2) reasoning about everyday problem definitions. Specifically, in this case we focused on the participant’s ability to consider contingencies or conditions such as hidden or underlying aspects.

#### Control group with problem-solving training

Participants in this group received standard materials derived from Artistico et al. (2010). The experimental procedure for this group was essentially identical to the procedure described above, but instead of focusing on exercise-related obstacles and solutions, we asked participants to work on a typical day-to-day problem (e.g., how to increase social contact with others, or how to cope with feelings of separation from a partner). Specifically, in Part A participants were presented with a list of solutions related to solving interpersonal problems of a day-to-day nature. There were 33 solutions for each of the problems presented (three problems total), and they were normatively the same for each participant. In Part B participants were presented with plausible root causes of the problems from Part A. In Part C the goal was to link solutions to root causes. Finally, in Part D the participants recounted out loud (or in writing) as many solutions as possible for the same problem.

#### Control group (without problem-solving training)

Participants in the control group did not receive any information on how to overcome their obstacles to exercise. Participants in this group were asked to simply complete measures related to self-efficacy for engaging in physical activity.

### Measures

All the measures were presented at baseline and only the Self-Efficacy to Exercise (SEE) scales were re-presented during the post treatment procedure. The demographic questions were presented at the end of the study.

#### Obstacles to exercise

The Obstacles to Exercise Survey (OES) was developed to identify participants’ primary reasons for not exercising. The OES is a self-administered questionnaire that consists of a series of questions aimed at identifying the participant’s three primary reasons for not exercising (e.g., lack of motivation, low energy). Specifically the OES is comprised of 18 questions that help the participant identify and explore challenges related to exercise engagement (e.g., have you attempted in the past to overcome the reason or problem that makes it difficult for you to exercise?). The perceived difficulty of the problem is also assessed using a 1–10 scale. The internal consistency of the challenge posed by the three problems was calculated (α = 0.86).

#### Self-efficacy to exercise scales

We also developed the SEE Scales. The SEE is a six-scale survey that measures an individual’s expectations of his or her self-efficacy when faced with barriers to exercise. For five of the scales (brisk walking, running, cycling, swimming, lifting weights), the participant responds on a scale from 1 (“You cannot accomplish the specific behaviors described”) to 10 (“You can certainly accomplish the specific behaviors described”) in order to describe their current level of confidence that they could exercise for various lengths of time or intensity (I can run 1–3 miles a week or I can run 1–3 miles everyday). The sixth scale on the survey asks participants to write in one activity that is their preferred way to exercise and then to rate that item on the same scale used for the previous five scales. To measure changes in self-efficacy, this measure was employed in both laboratory sessions. SEE scales were developed by closely following Bandura’s (1997) guiding principles. The coefficient of reliability α of the SEE ranged from 0.89 to 0.95 (session 1) and α = 0.90 to α = 0.95 (session 2).

#### Perceived social support

Perceived social support was assessed using the Social Provisions Inventory (SPI – Cutrona and Russell, 1987), which consists of 24 questions that assess an individual’s relationship with other people based on a four-point scale (Strongly disagree, Disagree, Agree, and Strongly agree). High numbers on this scale indicate satisfactory levels of perceived social support. The sub-dimensions of the SPI were computed to assess the SPI internal consistency (α = 0.86).

#### Perceived stress

Perceived stress was assessed with the Perceived Stress Scale (Cohen and Williamson, 1988), which consists of four questions that rate an individual’s feelings and thoughts during the past month based on a five-point scale (Never, Almost never, Sometimes, Fairly often, and Very often). High numbers on this scale indicate the presence of stress. We assessed the internal consistency (α = 0.84) of the scale in our sample.

#### Other measures

The experimenter completed the screening by interviewing the participants about the time elapsed since they exercised regularly (at least 4 months ago), and about the intention to exercise in the near future (yes/no). A standard questionnaire was administered to all participants to assess demographic characteristics such as gender, age, and race.

### Statistical analyses

A mixed between-within factorial design was implemented to test our main hypothesis. The three experimental groups comprised the *between* factor, with time (baseline and post-intervention) as the *within* factor. The primary dependent variables were levels of perceived self-efficacy for exercise at both time points. To test our main hypothesis, we conducted a repeated measures MANOVA where the within factor was represented by the assessment pre- and post-training, and the between factor was represented by the three different study groups.

## Results

### Baseline results and preliminary analysis

No significant differences (computed with a MANOVA) were observed among study groups on obstacles to exercise, self-efficacy, perceived social support, perceived stress (Table 1). At the outset of the analysis we correlated all the specific self-efficacy scales (running, brisk walking, swimming, lifting weights, and cycling). The “other activities” self-efficacy scale was not analyzed because the participants’ responses varied greatly from person to person (e.g., yoga, rock-climbing, dancing, etc.). The results indicated that all the scales were inter-correlated. The minimum correlation was *r* = 0.35, *p* < 0.05; and the maximum correlation was *r* = 0.78; *p* < 0.001.

**Table 1:** **Assessment of the three groups regarding selection criteria, background characteristics, and self-efficacy for exercise before training**.

Variables	Measure units	Groups			*P*-levels
		Experimental *M* (SD)	Control Training *M* (SD)	Control No training *M* (SD)
Obstacles to exercise	(Not challenging/challenging 1–10)	6.85 (1.85)	6.48 (1.43)	6.33 (0.99)	0.58
Self-efficacy	(I cannot/can exercise 1–10)	5.44 (1.49)	5.73 (2.07)	5.17 (2.18)	0.68
Perceived stress	(Never/always 0–4)	1.57 (0.87)	1.73 (0.83)	2.07 (1.03)	0.28
Perceived support	(Not supported/supported 1–4)	3.43 (0.26)	3.27 (0.34)	3.45 (0.35)	0.16

As part of the preliminary analysis, we looked at the obstacles to exercise reported by participants across study conditions. We computed frequencies by combining nuances of the same problems. For example time pressure when deciding to exercise (too much work, or not enough hours in a day, or too much homework) was reported by 50.3% of participants (each participant reported three obstacles) in the total obstacle count, followed by lack of energy (14.5%), feelings of shyness (I feel shy at the gym) or inadequacy (not too well coordinated) combined (12.7%), lack of intrinsic (i.e., exercising is boring) or extrinsic motivation (i.e., do not see the benefits of exercising) combined (9%) or social support (6%) from family and friends (i.e., I do not like to exercise alone), and inability to find a suitable space to work out (about 3%) plus other reasons (i.e., never played sports in my life). The correlation between the perceived challenge posed by the obstacles to exercise (aggregate score of the three obstacles reported) and the perceived self-efficacy for exercise was significant (*r* = −0.39; *p* < 0.01) with a negative valance – the more the perception of the challenge, the less the sense of self-efficacy for exercise.

### Main results

The multivariate tests confirmed the hypothesized interaction. The interaction was between the condition (experimental, control with training, control without training) and the time of assessment of the SEE scales (before and after the training). The significant effect of the interaction was driven by an increase in perceived self-efficacy in the experimental group with *F*_2,52_ = 4.98 (*p* < 0.02, η^2^ = 0.16).

**Figure 1:**
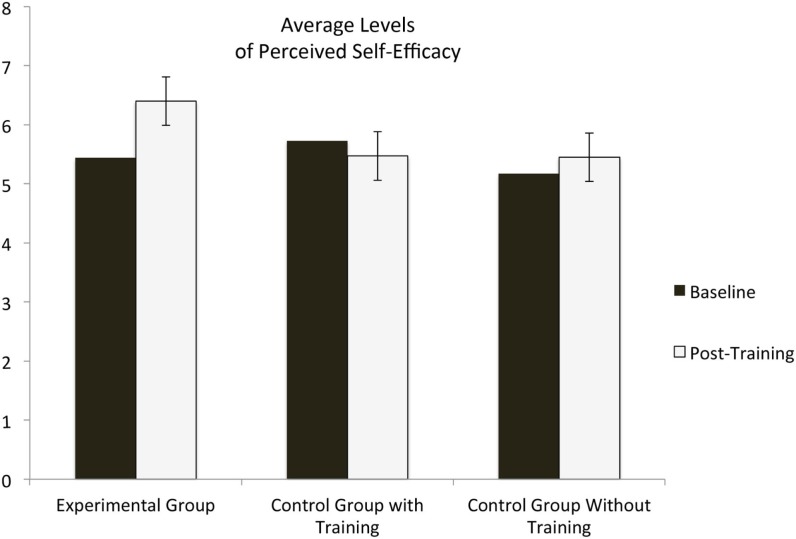
**It depicts average levels of self-efficacy across the three groups before and after the intervention**.

This increase in self-efficacy by the experimental group at post-intervention was significantly greater than the increase in self-efficacy in the other two groups. Specifically, the mean level of perceived self-efficacy was higher in the experimental group than in the control group with training: *t*_38_ = 2.95; *p* < 0.01. Also the self-efficacy mean level of the participants in the experimental group was greater than the one of the control group: *t*_31_ = 2.49, *p* < 0.02. Within group analysis showed that participants in the experimental group reported almost a standard deviation increase with respect to baseline (*t*_17_ = −3.35, *p* < 0.003): their post-training self-efficacy was *M* = 6.40 (SD = 1.04). Mean levels of self-efficacy of participants in the other two conditions (control with training, *t*_21_ = 1.25, *p* = 0.23, or control *t*_14_ = −0.73, *p* = 0.48) did not change significantly after the intervention. In Figure 1, we depicted the average perceived self-efficacy levels for the three groups as a function of treatment (pre- or post-intervention).

### Covariates and other analysis

We correlated self-efficacy scores before and after the procedure with perceived stress (*r* = −0.22) and perceived social support (*r* = 0.16): these correlations were not significant. Additionally, we did not find any significant co-variation in perceived stress (*F*_1,50_ = 0.43, ns) or social support (*F*_1,50_ = 1.88, ns), with self-efficacy measured before or after the intervention. Table 2 shows solutions that were chosen most frequently by the participants in the experimental group.

**Table 2:** **Most frequently chosen solutions for every day obstacles that hinder exercise**.

Solution	Times offered	Times chosen	%
	*n*	*n*	
Ask people you know who exercise to let you know when they are going and ask if you can join them	7	5	71
Be aware the exercise can be done anywhere, including small spaces or in your home	11	8	73
Before joining a gym, explain your situation to the gym manager or a trainer to see what solutions they might propose; make a commitment only to the facility that meets your approval	5	4	80
Exercise with a partner. Come up with rewards together (e.g., go to a favorite common restaurant after a good workout)	7	5	71
Consider that exercising with people you like could be motivating because it is more fun	5	5	100
Consider that the exercise routine will take time to show the benefits (e.g., improved mood, higher overall energy level)	6	6	100
Consider that your physical appearance will improve over time	6	6	100
Diversify your exercise routine with your friends so that it does not get boring	7	5	71
Do push-ups and sit ups when at home watching T.V., even if it’s only a few of each every hour or so	14	10	71
Improve your sleep habits by keeping on a regular sleep schedule	15	11	73
Listen to music while you exercise	23	20	87
Exercise with a partner. Make a compromise on the type of exercise, so that you both get to do your favorite activities	7	6	86
Note that there are more than just physical benefits to exercise (e.g., increased energy, increased happiness, increased focus)	5	4	80
Note the nutrition facts of foods and beverages you regularly eat and drink	14	10	71
Remind yourself that this effort and hard work is for your own benefit, not anyone else	21	16	76
Take advantage of weekends or other days off of work or school to exercise	14	10	71
Try to choose foods and beverages that are low in sugar, sodium, and fat, and are high in protein, vitamins, and minerals (e.g., vitamin C, calcium)	15	11	73

## Discussion

The results clearly indicated that it is possible to increase self-efficacy for exercise by improving everyday problem-solving ability. This was the goal of the study. Specifically, we achieved our goal by enhancing participants’ understanding of the obstacles or barriers that had been interfering with their intended exercise pursuits. The experimental procedure was tailored in order to capture within-person variability in participants’ perceived SEE. Each person may have reported differently similar problems or different problems altogether. Our work was designed to capitalize on these individual differences. The data showed that challenges varied from person to person in subtle ways. For example, a person might have reported time as being her biggest challenge because of family obligations whereas another person was unable to set time aside from school or friends.

The importance of analyzing several strategies to exercise obstacles was an asset of the new experimental procedure. Participants were guided to think divergently about their problems by exploring alternative solutions. Solution generation was proposed as a way to increase problem-solving, but the implementation of the solution was carefully linked to possible root causes of the problem. In everyday problem-solving theory, the link between solution generation and identification of root causes of the problem is considered one of the best ways to approach ill-defined problems.

Other investigators have also studied well-defined problems. The intent of well-defined everyday problem-solving research is to study the logical generation of solutions that can be considered optimal because all the nuances of the problems could be eliminated or controlled (Diehl et al., 2005; Willis et al., 2006). Here we were concerned mainly with such nuances. The point of the study was to show that even if problems are nominally the same (time pressure), the nuances of the problem can be addressed at the individual level rather than at the group level. We, instead of treating the problem “time” as the same for everybody, offered a tailored problem-solving strategy to each participant.

The results also indicated that the experimental manipulation was strong in comparison to other types of everyday problem-solving. For instance, work by Artistico et al. (2010), found a large number of solutions for problems that were relevant to younger adults (e.g., break-up with a boyfriend). When we applied the same solutions to ensure internal validity to the study (recall that the participants in the control group were exposed to these problem-solving strategies), participants in the control group did not show a significant increase in their self-efficacy for exercise. This null result documents that there was no task demand in our experiment; information learned by our participants in the control conditions did not translate into an increased self-efficacy in the exercise domain.

The magnitude of the change in self-efficacy reported by the experimental group was notable. Although measurement of behavior change (i.e., engagement in physical activity) was beyond the scope of this study, these results, in combination with the documented relationship between self-efficacy and behavior change, provide preliminary evidence for examining the utility of a problem-solving focused training in the context of a physical activity intervention. Changes in self-efficacy offer insights into the next step, that is, to follow through a designed action (Bandura, 1997). Our newly developed SEE scales “speak about” real exercise activities such as brisk walking. For example, brisk walking could be taught in a physical activity program.

Our study naturally contains limitations, including sample size, scope, and age of the participants. The sample comes from a similar educational background, where the external resources are the same (i.e., free gym on campus), thus the challenge of exercising could pose slightly different motivational demands. The age of the participants, scope of the study, and sample size could be addressed within a research plan that targets a more heterogeneous class of individuals. This research is in fact a germinal step toward the development of a large-scale analysis of one’s ability to overcome barriers and psychological blocks when entering an exercise routine. It will be useful to replicate these findings with an older population coming from a more experientially diverse background. As stated in the specialized literature on everyday problem-solving (see Blanchard-Fields, 2007 for a quick overview), age differences are noteworthy in everyday problem-solving ability. Also, because of the limited scope of the study (individual change in perceived self-efficacy), we did not assess the health status of the participants. This limitation can be overcome in a behavioral modification study where health indicators such as a body mass index are typically measured.

Despite the limitations, the study possesses strengths. For one, we experimentally increased perception of self-efficacy via everyday problem-solving ability in the domain of exercise. We as well other researchers obtained similar correlational findings in the past (Artistico et al., 2003; Pezzuti et al., 2009), but never studied the individual’s perception of self-efficacy for exercise as a “dependent variable.” Thus, the findings are novel. Second, we intended to measure shifts in people’s confidence in their ability to exercise through a systematic approach to problem-solving (the systematic approach is the cognition of mental efforts to a practical sense of being able to exercise). Although not measured directly in our study, research on health intervention has documented that self-efficacy perceptions predict behavioral modification in a variety of clinical settings.

Applied research could benefit from the development of these findings. For instance, given the known beneficial effects of physical exercise, it would be useful to apply panel study methods to foster knowledge learned during the lab procedures in real life scenarios. The vision for the future is a longitudinal study where the objective is to capitalize on individual differences (at the individual level) and not just group differences regarding self-efficacy, everyday problem-solving, and exercise. Generating solutions and reasoning about everyday problem definitions can be used as the foci of intervention procedures where the goal is to provide people with alternative ways of achieving health promotion via self-efficacy for exercise.

## Conflict of Interest Statement

The authors declare that the research was conducted in the absence of any commercial or financial relationships that could be construed as a potential conflict of interest.

## References

[B1] Allaire J. C. Marsiske M. (2002). Well- and ill-defined measures of everyday cognition: relationship to elders’ intellectual ability and functional status. Psychol. Aging 17, 101–115 10.1037/0882-7974.17.1.101 11931279 PMC2909873

[B2] Artistico D. Berry J. Black J. Cervone D. Lee L. Orom H. (2011). “Psychological functioning in older age: a self-efficacy analysis,” in The Oxford Handbook of Reciprocal Adult Development and Learning, ed. Hoare C. H. (New York: Oxford University Press), 215–247

[B3] Artistico D. Cervone D. Pezzuti L. (2003). Perceived self-efficacy and everyday problem solving among young and older adults. Psychol. Aging 18, 68–79 10.1037/0882-7974.18.1.68 12641313

[B4] Artistico D. Orom H. Cervone D. Krausse S. Houston E. (2010). Everyday challenges: the influence of contextual factors on everyday problem solving among young, middle-aged, and older adults. Exp. Aging Res. 36, 230–247 10.1080/03610731003613938 20209423

[B5] Bandura A. (1977). Self-efficacy: toward a unifying theory of behavioral change. Psychol. Rev. 84, 191–215 10.1037/0033-295X.84.2.191 847061

[B6] Bandura A. (1986). Social Foundation of Thoughts and Action: A Social Cognitive Theory. Englewood Cliffs, NJ: Prentice-Hall

[B7] Bandura A. (1997). Self-Efficacy: The Exercise of Control. New York: Freeman and Company

[B8] Bandura A. Cervone D. (1983). Self-evaluative and self-efficacy mechanisms governing the motivational effects of goal systems. J. Pers. Soc. Psychol. 45, 1017–1028 10.1037/0022-3514.45.5.1017

[B9] Blanchard-Fields F. (2007). Everyday problem solving and emotion: an adult developmental perspective. Curr. Dir. Psychol. Sci. 16, 26–31 10.1111/j.1467-8721.2007.00469.x

[B10] Bureau of Labor Statistics (2009). Sports and Exercise. Available at: http://www.bls.gov/spotlight/2008/sports/

[B11] Caprara G. V. Cervone D. (2000). Personality: Determinants, Dynamics, and Potentials. Cambridge: Cambridge University Press

[B12] Cervone D. (2004). The architecture of personality. Psychol. Rev. 111, 184–203 10.1037/0033-295X.111.1.183 14756593

[B13] Cohen S. Williamson G. (1988). “Perceived stress in a probability sample of the U.S.,” in The Social Psychology of Health: Claremont Symposium on Applied Social Psychology, eds Spacapam S. Oskamp S. (Newbury Park, CA: Sage), 31–67

[B14] Colcombe S. J. Kramer A. F. Erickson K. I. Scalf P. McAuley E. Cohen N. J. (2004). Cardiovascular fitness, cortical plasticity, and aging. Proc. Natl. Acad. Sci. U.S.A. 101, 3316–3321 10.1073/pnas.0400266101 14978288 PMC373255

[B15] Cutrona C. E. Russell D. (1987). “The provisions of social relationships and adaptation to stress,” in Advances in Personal Relationships, Vol. 1, eds Jones W. H. Perlman D. (Greenwich, CT: JAI Press), 37–67

[B16] Diehl M. Marsiske M. Horgas A. L. Rosenberg A. Saczynski J. S. Willis S. L. (2005). Revised observed tasks of daily living: a performance-based assessment of everyday problem-solving in older adults. J. Appl. Gerontol. 24, 211–230 10.1177/0733464804273772 18160968 PMC2153442

[B17] Dishman R. K. Motl R. W. Saunders R. P. Felton G. Ward D. S. Dowda M. (2004). Self-efficacy partially mediates the effect of a school-based physical-activity intervention among adolescent girls. Prev. Med. 38, 100–108 10.1016/j.ypmed.2003.12.007 15066366

[B18] Dishman R. K. Saunders R. P. Motl R. W. Dowda M. Pate R. R. (2009). Self-efficacy moderates the relation between declines in physical activity and perceived social support in high school girls. J. Pediatr. Psychol. 34, 441–451 10.1093/jpepsy/jsn100 18812410 PMC2671981

[B19] Doerksen S. E. Umstattd M. R. McAuley E. (2009). Social cognitive determinants of moderate and vigorous physical activity in college freshmen. J. Appl. Soc. Psychol. 39, 1201–1213 10.1111/j.1559-1816.2009.00478.x

[B20] Flöel A. Ruscheweyh R. Krüger K. Willemer C. Winter B. Völker K. (2009). Physical activity and memory functions: are neurotrophins and cerebral gray matter volume the missing link? Neuroimage 49, 2756–2763 19853041 10.1016/j.neuroimage.2009.10.043

[B21] Hamman R. F. Wing R. R. Edelstein S. L. Lachin J. M. Bray G. A. Delahanty L. (2006). Effect of weight loss with lifestyle intervention on risk of diabetes. Diabetes Care 29, 2102–2107 10.2337/dc06-0560 16936160 PMC1762038

[B22] Keppel G. (1991). Design and Analysis: A Researcher’s Handbook. Upper Saddle River, NJ: Prentice-Hall, Inc.

[B23] Lee L. Arthur A. Avis M. (2008). Using self- efficacy theory to develop interventions that help older people overcome psychological barriers to physical activity: a discussion paper. Int. J. Nurs. Stud. 45, 1690–1699 10.1016/j.ijnurstu.2007.05.008 18501359

[B24] Pezzuti L. Artistico D. Cervone D. Tramitolo C. Black J. (2009). Assessing the efficacy of a brief everyday problem solving training program for older adults. TPM 16, 111–127

[B25] Plotnikoff R. C. Higginbotham N. (2002). Protection motivation theory and exercise behavior change for the prevention of coronary heart disease in a high-risk, Australian representative community sample of adults. Psychol. Health Med. 7, 87–98 10.1080/13548500120101586

[B26] Prohaska T. R. Peters K. Warren J. S. (2000). Sources of attrition in a church-based exercise program for older African-Americans. Am. J. Health Promot. 14, 380–385 10.4278/0890-1171-14.6.380 11067573

[B27] Reddigan J. I. Ardern C. I. Riddell M. C. Kuk J. L. (2011). Relation of physical activity to cardiovascular disease mortality and the influence of cardiometabolic risk factors. Am. J. Cardiol. 108, 1426–1431 10.1016/j.amjcard.2011.07.005 21855834

[B28] Reitman W. R. (1964). Heuristic Decision Procedures Open Constraints and the Structure of Ill-Defined Problems, Chap. 15. New York: John Wiley & Sons Inc., 282–315

[B29] Roessler K. K. Ibsen B. (2009). Promoting exercise on prescription: recruitment, motivation, barriers and adherence in a Danish community intervention study to reduce type 2 diabetes, dyslipidemia and hypertension. J. Public Health (Bangkok) 17, 187–193 10.1007/s10389-008-0235-4

[B30] Simon H. A. (1973). The structure of ill-structured problems. Artif. Intell. 4, 181–201 10.1016/0004-3702(73)90011-8

[B31] Walcott-McQuigg J. A. Prohaska T. R. (2001). Factors influencing participation of African American elders in exercise behavior. Public Health Nurs. 18, 194–203 10.1046/j.1525-1446.2001.00194.x 11359621

[B32] Warburton D. E. Nicol C. W. Bredin S. S. (2006). Health benefits of physical activity: the evidence. Can. Med. Assoc. J. 174, 801–809 10.1503/cmaj.051351 16534088 PMC1402378

[B33] Willis S. L. Tennstedt S. L. Marsiske M. Ball K. Elias J. Koepke K. M. (2006). Long-term effects of cognitive training on everyday functional outcomes in older adults. JAMA 296, 2805–2814 10.1001/jama.296.23.2805 17179457 PMC2910591

